# Announcement: Chemistry, Biochemistry and Therapeutic Applications of Vanadium Compounds

**DOI:** 10.1155/MBD.1997.247

**Published:** 1997


					THE FIFTH CHEMICAL CONGRESS OF NORTH AMERICA
November 11-15, 1997
Cancun, Quintana Roo, Mexico
SYMPOSIUM" CHEMISTRY, BIOCHEMISTRY
AND THERAPEUTIC APPLICATIONS OF VANADIUM COMPOUNDS
FINAL PROGRAM
Wednesday Morning, Peter Schwendt, Presiding
9:00 a.m. F.H. Nielsen
The Nutritional Essentiality and Physiological Metabolism of Vanadium in Higher Animals
9:30 a.m. L. Pettersson
Studies of Vanadate-Organic Ligand Systems Using Potentiometry and NMR Spectroscopy
10:00 a.m. D. Rehder, M. Bashirpoor, S. Jantzen, H. Schmidt
Structural and Functional Models for Biogenic Vanadium Compounds
10:30 a.m. C.R. Cornman, T. C. Stauffer, P. D. Boyle
Synthesis, Structure and Reactivity of V(V)-Thiolate and V(V)-eta2-Sulfenate Complexes
10:50 a.m. T. Hirao
Selective Synthetic Transformations via Vanadium-Induced Redox
11:10 a.m. Intermission
11:20 a.m. S. Chen, H. Ruetthard, A. Banerjee, F. S. Jiang, M. Sprinzi, M. W. Makinen
Vanadyl Cation as a Probe of Nucleotide and Protein Structure
11:50 a.m. J. Petersen, T. R. Hawkes, D. J. Lowe
The Vanadyl Coordination Environment in Imidazole Glycerol Phosphate Dehydratase
12:10 p.m. H. Michibata, T. Uyama, K. Kanamori
The Accumulation Mechanism of Vanadium by Ascidians
Wednesday Afternoon, J. Antonio Guevara-Garcia, Presiding
3:30 p.m. A. Messerschmidt, L. Prade, R. Wever
Chloroperoxidase from Curvularia inaequalis: X-Ray Structures of Native and Peroxide Form Reveal
Vanadium Chemistry in Vanadium Haloperoxidases
3:50 p.m. C. Slebodnick, M. E. Gillis, B. J. Hamstra, V. L. Pecoraro
Peroxidase Activity of Vanadium Haloperoxidase Model Compounds
4:20 p.m. A. Butler, A. Baldwin, M. Simpson
Selectivity of Vanadium Bromoperoxidase with Indole Derivatives
4:50 p.m. R. Wever, W. Hemrika, R. Renirie, P. Barnett, A. Messerschmidt, Henk Dekker
From Vanadium Haloperoxidases to Phosphatases, the Same Architecture for the Active Site
5:10 p.m. Intermission
5:20 p.m. F. Nxumalo, A. S. Tracey
A New Class of Insulin-Mimetic Compounds: N,N-Dimethylhydroxamidovanadates, Aspects of Their
Chemistry and Function
5:50 p.m. D.C. Crans
New Peroxo- and Hydroxylamino Vanadium Complexes: Chemistry, Phosphatase Activity and
Insulin Mimetic Properties
6:20 p.m. I.G. Fantus, B. Lu
Paradoxical Enhanced Sensitivity of Insulin Resistant Adipocytes to Vanadate is Associated with
Decreased Intracellular Reduction of Vanadate(+5) to Vanadyl(+4)
6:40 p.m. S.K. Pandey, M. B. Anand-Srivastava, A. K. Srivastava
Vanadyl Sulfate (VS)-Induced Glycogen Synthesis in Chinese Hamster Ovary Cells Over-
expressing Human Insulin Receptor (CHO-HIR Cells) is Blocked by Wortmannin and LY294002,
Inhibitors of Phosphatidyl Inositol 3-Kinase (PI3-K)
Thursday Morning, Debbie C. Crans, Presiding
9:00 a.m. C. Orvig, J. H. McNeill
Coordination Chemistry of Insulin-Mimetic Vanadium Compounds
9:30 a.m. Y. Shechter, E. Elberg, N. Sekar, Z.-B. He, D. Gefel, J. Meyerovitch, R. Bruck, E.
Gershonov, A. Shishevia, D. C. Crans, R. Sigar, M. Fridkin, Y. Goldwasser, J. Li
On the Insulin-Like Effects of Vanadium Salts
10:00 a.m. Intermission
10:10 a.m. S.B. Etcheverry, A. M. Cortizo
Vanadium Bioactivity on Cells in Culture
247
10:40 a.m. B.I. Posner, A. Shaver
Mechanism of Action of Peroxovanadium Compounds
11:10 a.m. H. Sakurai
Structure-Activity Relationships of the Insulin-Mimetic Vanadyl Complexes
11:40 a.m. G.R. Willsky, A. B. Goldfine, C. R. Kahn, P. J. Kostyniak
Pharmacokinetics of Vanadium Following Repeated Oral Dosing with Vanadyl Sulfate in Patients
with NIDDM.
12:00 p.m. C.R. Kahn, A. B. Goldfine
Vanadium Salts in the Treatment or Human Diabetes Mellitus.
Wednesday Evening, Oral Poster session, Alan S. Tracey, Presiding
7:30 10:00 p.m.
J. J. Cruywagen
Vanadium(V) Equilibria: Stability Constants and Thermodynamic Quantities for the Various Ionic
Species
D. C. Crans, F. Xin
Estimating Formation Constants for Vanadium(V)-Pentose Complexes.
M. Rangel, M. Castro, W. Schlindwein, R. Matias, B. Castro, C. Geraldes, J. Burgess
Solution Chemistry of Bis(1,2-dimethyl-3-hydroxy-4-pyridinonate)oxo-vanadium(IV) in Water
C. R. Cornman, K. M. Geiser-Bush, P. D. Boyle
Synthesis and Characterization of Trigonal Bipyramidal Vanadyl Complexes: Structural and
Electronic Constants on Molecular Geometry.
D. C. Crans, B. Zhang Reactions of Vanadate with Thiol Compounds: Complex Formation and
Redox Chemistry.
P. Schwendt, M. Sivak Composition and Structure of Vanadium(V) Peroxo Complexes
V. Conte, F. Di Furia, S. Moro
From the Speciation of Peroxovanadium Complexes in Aqueous Solution to the Chemistry of
Haloperoxidases
J. A. Guevara-Garcia, G. Mendoza-Diaz and N. Barba-Behrens
Bis-peroxo-oxovanadium(V) Complexes of Histidine-Containing Peptides as Models for Vanadium
Halo-peroxidases
C. Slebodnick, G. J. Colpas, V. L. Pecoraro
Vanadium Chloroperoxidase Models: Solvent Dependence of Chloride Oxidation Rates by
Vanadate
I. W. Arends, R. A. Sheldon
Catalytic Oxidations with Biomimetic Vanadium Systems
H. B. ten Brink, W. Hemrika, H. L. Dekker, H. E. Schoemaker, R. Wever
The Applications of Vanadium Peroxidases as Novel Biocatalysts
R. Renirie, W. Hemrika, H. L. Dekker, P. Barnett, R. Wever, E. C. Slater
Exploring the Active Site of Vanadium Chloroperoxidase
C. M. M. Matoso, A. J. L. Pombeiro, J. J. R. Frausto da Silva, M. F. C. Guedes
da Silva, J. A. L. Silva
A Possible Role for Amavadine in Some Amanita Fungi
G. R. Willsky, D. C. Crans
What is the Active Vanadium Species Inhibiting Growth of S. cerevisiae in High Phosphate Growth
Medium?
I. Nieves-Martinez, C. J. Olivo-Delgado, R. P. Mason
Determination of the Vanadate-Mediated NAD(P)H or NADH Oxidation Mechanism by Superoxide
Radical
C. F. G. C. Geraldes, M. M. C. A. Castro, R. Ramasamy, A. D. Sherry
Influence of Vanadate on Glycolysis, Intracellular Sodium and pH in Perfused Rat Hearts.
I. G. Fantus, E. Tsiani, A. Sorisky
Vanadate and Pervanadate Stimulate Glucose Uptake in L6 Skeletal Muscle Cells by a Mechanism
Independent on Phosphatidylinositol 3-Kinase and Protein Kinase.
J. A. Guevara-Garcia, E. B. Colombres, E. Gonzalez-Vergara, R. Tapia-Benavides, C. E. Weinmann
New Vanadium Compounds in The Treatment of Diabetes-Induced Rats.
For complementary information, please contact
Alan S. Tracey
Department of Chemistry and Institute of Molecular Biology and Biochemistry
Simon Fraser University
Burnaby, B.C., V5A 1S6, Canada
Tel (604) 291 4464, Fax (604) 291 3765
248

				

